# Markers of thrombin generation are associated with myocardial necrosis and left ventricular impairment in patients with ST-elevation myocardial infarction

**DOI:** 10.1186/s12959-015-0061-1

**Published:** 2015-09-22

**Authors:** C. H. Hansen, V. Ritschel, S. Halvorsen, G. Ø. Andersen, R. Bjørnerheim, J. Eritsland, H. Arnesen, I. Seljeflot

**Affiliations:** Center for Clinical Heart Research, Oslo University Hospital Ullevål, PB 4956 Nydalen, N-4956 Oslo, Norway; Departement of Cardiology, Oslo University Hospital Ullevål, Oslo, Norway; Faculty of Medicine, University of Oslo, Oslo, Norway

**Keywords:** Myocardial infarction, D-dimer, Prothrombin fragment 1 + 2, Myocardial function

## Abstract

**Introduction:**

Platelet activation, thrombin generation and fibrin formation play important roles in intracoronary thrombus formation, which may lead to acute myocardial infarction.

We investigated whether the prothrombotic markers D-dimer, pro-thrombin fragment 1 + 2 (F1 + 2) and endogenous thrombin potential (ETP) are associated with myocardial necrosis assessed by Troponin T (TnT), and left ventricular impairment assessed by left ventricular ejection fraction (LVEF) and N-terminal pro b-type natriuretic peptide (NT-proBNP).

**Materials/Methods:**

Patients (*n* = 987) with ST-elevation mycardial infarction (STEMI) were included. Blood samples were drawn at a median time of 24 h after onset of symptoms.

**Results:**

Statistically significant correlations were found between both peak TnT and D-dimer (*p* < 0.001) and F1 + 2 (*p* < 0.001), and between NT-proBNP and D-dimer (*p* = 0.001) and F1 + 2 (*p* < 0.001). When dividing TnT and NT-proBNP levels into quartiles there were significant trends for increased levels of both markers across quartiles (all *p* < 0.001) D-dimer remained significantly associated with NT-proBNP after adjustments for covariates (*p* = 0.001) whereas the association between NTproBNP and F1 + 2 was no longer statistically significant (*p* = 0.324).

A significant inverse correlation was found between LVEF and D-dimer (*p* < 0.001) and F1 + 2 (*p* = 0.013). When dichotomizing LVEF levels at 40 %, we observed significantly higher levels of both D-dimer (*p* < 0.001) and F1 + 2 (*p* = 0.016) in the group with low EF (*n* = 147).

**Summary/conclusion:**

In our cohort of STEMI patients we demonstrated that levels of D-dimer and F1 + 2 were significantly associated with myocardial necrosis as assessed by peak TnT. High levels of these coagulation markers in patients with low LVEF and high NTproBNP may indicate a hypercoagulable state in patients with impaired myocardial function.

## Introduction

Coronary artery disease (CAD) is a progressive atherosclerotic condition and is, together with thrombus formation, the most important underlying mechanism of an acute myocardial infarction (AMI) [1–4].

In addition to platelet activation, thrombin generation and fibrin formation play an important role in the development of an intracoronary thrombus, which may lead to an acute coronary occlusion [2, 5, 6].

The generation of thrombin through the tissue factor pathway is essential in the haemostatic process [1, 2, 7]. It is crucial in normal physiology, whereas an inappropriate generation of thrombin may contribute to vascular occlusions such as in myocardial infarction. Increased thrombin generation, as an expression of activation of the coagulation system, was previously shown in patients with acute coronary syndrome and unstable angina pectoris [8, 9].

When prothrombin is converted to activated thrombin, prothrombin fragment 1 + 2 (F1 + 2) is formed, thus indicating thrombin generation *in vivo*, with subsequent fibrin formation. From the fibrinolytic system, plasmin converted from plasminogen degrades fibrin, resulting in degredation products like D-dimer. Elevated D-dimer levels therefore indicate both ongoing coagulation and fibrinolytic activation. Both markers have been shown to be persistantly elevated for months after the acute myocardial infarction [5], whereas an early decrease in D-dimer levels has been shown to be associated with improved prognosis [10].

The endogenous thrombin potential (ETP) has been proposed as an informative method to determine the degree of hypercoagulability, measuring the potential to generate thrombin *ex vivo* [11].

Several studies have shown increased levels of prothrombotic markers in patients with myocardial infarction [10, 12] and also association to clinical outcome [10, 13, 14]. There is, however, limited knowledge about activation of the coagulation cascade in the acute phase of ST-elevation myocardial infarction (STEMI) and also limited data on the degree of hypercoagulability in relation to the degree of myocardial injury and severity of the disease in these patients.

The aim of the present substudy was therefore to investigate whether circulating levels of selected prothrombotic markers were associated with the degree of myocardial necrosis assessed by peak troponin T (TnT) and with left ventricular impairment assessed by left ventricular ejection fraction (LVEF) and N-terminal pro b-type natriuretic peptide (NT-proBNP) in STEMI patients. Furthermore, the degree of hypercoagulability was studied in relation to traditional risk factors and baseline characteristics of the STEMI population.

## Material and methods

A total of 987 percutaneous coronary intervention (PCI)-treated STEMI patients from a cross sectional cohort study were included, all admitted to Oslo University Hospital Ullevål, Oslo, Norway in the period from June 2007 to August 2011. STEMI was defined as ST segment elevation of >2 mm in two or more contiguous chest leads or > 1 mm in two or more limb leads or left bundle branch block, together with typical chest pain and elevated troponin levels above the recommended diagnostic threshold. Patients on warfarin treatment, below 18 years of age and patients unable or unwilling to give written informed consent were excluded. Blood samples were collected at median time of 24 h after symptoms and 18 h after the PCI procedure, between 8 and 10 a.m. the following morning. In order to standardize blood sampling, and also to avoid any influence of diurnal variations and food intake, all samples were taken after an overnight fast. Routine blood samples were drawn at hospital admission, and samples for TnT were measured after standardized time intervals.

Citrated blood (0.129 M trisodium citrate in dilution 1:10) was centrifuged within 30 min at 2500 × g at 4 °C and kept frozen at ÷80 °C until analyzed. D-dimer and F1 + 2 were determined by ELISA (Asserachrom D-dimer, Stago Diagnostica, Ansiere, France and Enzygnost F1 + 2, Siemens, Marburg, Germany, respectively). Coefficients of variation (CV) were for D-dimer 6.5 % and F1+ 2 5.4 %.

ETP was determined by the Calibrated Automated Thrombogram (CAT) assay according to the manufacturer’s instructions (Thrombinoscope BV, Maastricht, The Netherlands) and thrombin generation was measured on the Fluoroscan Ascent fluorometer (Thermo Fisher Scientific OY, Vantaa, Finland). A reagent mixture of rTF and phospholipids in addition to a thrombin-specific fluorogenic substrate in Hepes buffer containing CaCl_2_ was added to the plasma to obtain a final concentration of 5 pM, 4 μM and 416.7 μM, respectively. In order to calculate the final results, plasma was measured along with a thrombin calibrator. The software (version 3.0.0.29; Thrombinoscope BV) enabled the calculation of the lag time (LT), peak thrombin generation (pTG), ETP and time to peak (TTP). Further, V_T_ (Velocity Index) = TP/(TTP-LT), indicating the average net rate of prothrombin activation during the propagation phase, was calculated. All experiments were run in duplicates and the interassay coefficients of variation for the different CAT parameters were 14.2, 4.6, 5.0 and 8.0 %, respectively.

CRP was measured with kits from DRG Instruments (Marburg/Lahn, Germany), CV <5 %.

Electrochemiluminescence technology for quantitative measurement was used for repeated measures of TnT (3rd generation cTroponinT, Elecsys 2010, Roche, Mannheim, Germany). The lower detection limit of the assay is 10 ng/L with a recommended diagnostics threshold of 30 ng/L. The inter-assay coefficient of variation was 7 %. NT-ProBNP was measured in serum using Elecsys proBNP sandwich immunoassay on Elecsys 2010 (Roche Diagnostics, Indianapolis, USA). The inter-assay coefficient of variation was 7 %.

Left ventricular ejection fraction (LVEF) was measured by echocardiography before hospital discharge or at a clinical follow-up within 3 months after the AMI (*n* = 767).

Diabetes was defined according to the American Diabetes Association criteria [15] and hypertension (HT) was defined as previously diagnosed and treated hypertension. Smokers were defined as current smokers or quit within the last month.

Clinical information was collected from hospital records and questionnaires acquired at the time of inclusion. Patients on warfarin were not included in this patient cohort.

The study was approved by The Regional Ethics Commitee and all patients gave written informed consent.

### Statistical analysis

Continous variables are presented as median values with 25,75 percentiles and categorial variables as number or proportions. As most of the variables were skewed, correlation analyses were performed using Spearman’s method. Differences between groups were tested by Mann–Whitney U test for continuous variables. Associations between prothrombotic markers and peak TnT and left ventricular impairment were tested in multivariate regression models, adjusting for relevant covariates. As the markers are strongly inter-related they were analyzed in separate models. Skewed data were log-transfomed before entered in the model. P values < 0.05 were considered statistically significant. The statistical analyses were performed with SPSS software version 18.0 (SPSS Inc, Chicago, USA).

## Results

Baseline characteristics of the total population are given in Table 1. The cohort was a typical STEMI population of relatively young, predominantly male patients (81 %) with medium size infarction (measured by peak TnT). Only 23 % with previous CVD, 12 % with known diabetes and half of the patients were smokers.

**Table 1:** Characteristics of the study population (*n* = 987)

Age (years) (range)	61 (24–94)
Male sex	800 (81)
Current smokers	474 (48)
Previous CVD	229 (23)
Treated hypertension	334 (34)
Treated diabetes mellitus	124 (12)
BMI (kg/m^2^)	26.6 (24.3,29.2)
Prehospital thrombolysis	119 (12)
Aspirin	231 (23)
Statins	233 (23)
Total cholesterol (mmol/L)	4.9 (4.1,5.6)
HDL (mmol/L)	1.06 (0.88,1.30)
Triglycerides (mmol/L)	1.25 (0.89,1.78)
CRP (mg/L)	13.4 (7.0,31.3)
Admission glucose (mmol/L)	7.4 (6.3,9.0)
Fasting glucose (mmol/L)	5.8 (5.2,6.6)
HbA1c (%)	5.9 (5.6,6.3)
Peak Troponin T (ng/L)	3850 (1710,7250)
NT-ProBNP (pmol/L)	31 (10,118)
LV Ejection fraction (%)	50 (44,55)
D-dimer (ng/mL)	456 (287,796)
F1 + 2 (pmol/L)	246 (178,356)
ETP (nM⋅min)	1564 (1366,1743)
Time from onset of symptoms to blood sampling (hours) (range)	24 (5–118)

Number (proportions) or median (25,75 percentiles) are given

*BMI* Body mass index, *CVD* Cardiovascular Disease, *HDL* High Density lipoprotein cholesterol, *CRP* C-reactive Protein, *ETP* endogenous thrombin potential

Levels of the haemostatic variables in the total population are shown in Table 1.

There were strong inter-correlations between D-dimer and F1 + 2 (*r* = 0.504, *p* < 0.001), and a weaker, inverse correlation between D-dimer and ETP (*r* = −0-.102, *p* < 0.001).

As visualized in Table 2, age was significantly correlated with F1 + 2 and D-dimer, inversely to ETP (all *p* < 0.001) and further weakly correlated to lag time, time to peak, peak hight and velocity index (all *p* < 0.05).

**Table 2:** Correlations between prothrombotic markers and selected variables

		D-dimer	F1 + 2	ETP	LT	TTP	pTG	V_T_
Age	r	0.412	0.277	−0.229	−0.075	−0.155	−0.086	0.088
	p	<0.001	<0.001	<0.001	0.021	<0.001	0.008	0.006
BMI	r	−0.184	−0.203	0.218	0.115	0.103	0.183	0.070
	p	<0.001	<0.001	<0.001	<0.001	0.001	<0.001	0.032
NT-ProBNP	r	0.243	0.120	−0.118	0.072	0.003	−0.033	0.066
	p	<0.001	<0.001	0.002	0.026	0.92	0.30	0.044
HbA1c	r	0.063	−0.015	0.033	0.025	0.006	0.055	0.048
	p	0.141	0.765	0.161	0.44	0.85	0.09	0.146
Fasting glucose	r	0.006	0.062	−0.054	0.042	−0.031	0.061	0.104
	p	0.844	0.074	0.115	0.19	0.34	0.06	0.001
Peak TnT	r	0.260	0.364	−0.072	−0.012	−0.059	0.001	0.065
	p	<0.001	<0.001	0.015	0.70	0.062	0.97	0.044
LVEF	r	−0.160	−0.090	0.022	−0.040	0.024	−0.065	−0.107
	p	<0.001	0.013	0.553	0.27	0.51	0.077	0.003

*TnT* Troponin T, *LVEF* left ventricular ejection fraction, *BMI* Body mass index, *LT* lag time, *TTP* Time to peak, *pTG* peak thrombin generation, *V*_*T*_ Velocity index

*r*-values refer to Spearman’s rank correlation coefficient

### Prothrombotic markers and association with myocardial injury

**Fig. 1:**
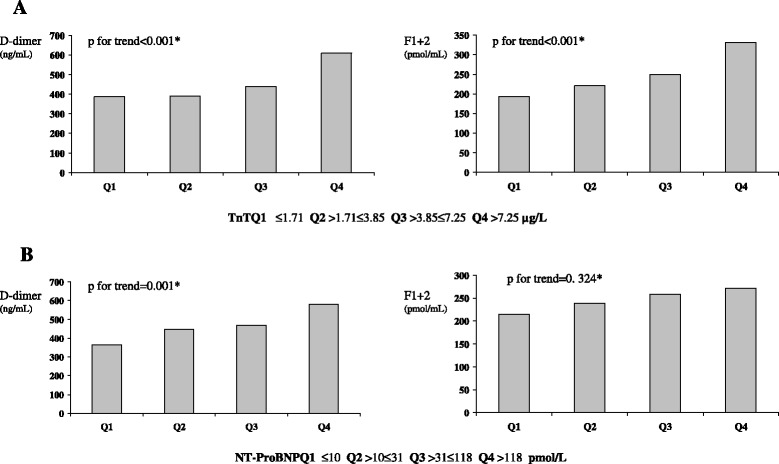
D-dimer and F1 + 2 (medians) in quartiles of peak TnT (**a**) and NT-ProBNP (**b**). **a** * = adjusted for age, sex, BMI, hypertension, time from symptoms to blood sampling, CRP and NT-ProBNP; **b** * = adjusted for age, sex, BMI, hypertension, time from symptoms to blood sampling and CRP

Statistically significant correlations were found between peak TnT and D-dimer and F1 + 2 (both *p* < 0.001) (Table 2). Linear trend analysis across quartiles of peak TnT revealed increased levels of both markers with increasing quartiles (p for trend < 0.001). When adjusting for relevant covariates as visualized in Table 4a both D-dimer and F1 + 2 remained significantly associated with peak TnT (both *p* < 0.001) (Table 4a and Fig. 1a). Weak, but statistically significant correlations were observed between TnT and ETP and velocity index (Table 2).

### Prothrombotic markers and association with myocardial function

Significant correlations were found between D-dimer and F1 + 2 and NT-ProBNP (both *p* = 0.001). Weak, but statistically significant correlations were also observed for the CAT-variables ETP, lagtime and velocity index (Table 2). When dividing NT-ProBNP levels into quartiles there were significant trends for increased levels of D-dimer and F1 + 2 across quartiles (both *p* < 0.001). D-dimer remained significantly associated with NT-proBNP after adjusting for covariates as visualized in Table 4b (*p* = 0.001), whereas the association between NT-proBNP and F1 + 2 was no longer statistically significant (*p* = 0.446) (Table 4b, Fig. 1b).

A weak, but significant inverse correlation was found between LVEF and D-dimer (*p* < 0.001), F1 + 2 (*p* = 0.013) and velocity index (*p* = 0.003) (Table 2). When dichotomizing LVEF levels at 40 % we observed significantly higher levels of all variables (*p* < 0.001, *p* = 0.016 and *p* = 0.004, respectively) in patients with LVEF below (*n* = 147), compared to above 40 % (Table 3). After adjustments for the covariates visualized in Table 4b the difference in D-dimer levels remained statistically significant (*p* = 0.003) whereas the association between LVEF and F1 + 2 and velocity index was no longer significant (*p* = 0.552 and *p* = 0.084, respectively).

**Table 3:** Levels of the prothrombotic markers according to group characteristics of the population

		n	D-dimer (ng/mL)	F1 + 2 (pmol/L)	ETP (nM⋅min)
Sex	Male	800	424 (275,755)	238 (175,350)	1576 (1401,1748)
	Female	187	609 (399,1008)	287 (203,395)	1494 (1284,1702)
	p		0.001	0.001	0.001
Smoking	+	474	448 (275,796)	245 (179,347)	1573 (1364,1762)
	-	513	467 (295,795)	250 (178,382)	1557 (1368,1726)
	p		0.675	0.594	0.269
Previous CVD	+	229	484 (286,798)	244 (177,332)	1569 (1348,1733)
	-	758	452 (286,796)	247 (180,369)	1563 (1370,1745)
	p		0.553	0.386	0.503
HT	+	553	553 (355,952)	275 (197,398)	1563 (1366,1730)
	-	418	418 (273,773)	235 (174,346)	1565 (1366,1745)
	p		0.001	0.001	0.381
Diabetes	+	870	454 (246,848)	232 (169,324)	1490 (1261,1754)
	-	117	456 (292,795)	249 (180,365)	1572 (1373,1754)
	p		0.285	0.142	0.004
LVEF (%)	>40	147	440 (285,755)	242 (174,363)	1573 (1371,1735)
	≤40	620	679 (408,1156)	297 (189,397)	1488 (1293,1691)
	p		<0.001	0.016	0.017

Median (25,75 percentiles) values are given

*HT* hypertension, *CVD* cardiovascular disease, *LVEF* left ventricular ejection fraction

**Table 4:** Determinants of peak Troponin T (a) and NT-proBNP (b)

Determinants	Standardized beta	95 % CI^c^	*p*-value	Determinants	Standardized beta	95 % CI	*p*-value
a)							
D-dimer	0.138	<0.001– < 0.001	<0.001	F1 + 2	0.216	0.001–0.001	<0.001
Age	−0.054	−0.012–0.002	0.156	Age	−0.052	−0.012–0.002	0.157
Sex	0.025	−0.117–0.261	0.457	Sex	0.020	−0.128–0.244	0.542
BMI	−0.026	−0.025–0.012	0.466	BMI	−0.013	−0.22–0.015	0.706
HT	−0.006	−0.179–0.152	0.870	HT	−0.013	−0.194–0.132	0.710
Time frame^a^	−0.076	−0.018– − 0.001	0.032	Time frame^a^	−0.071	−0.017– < −0.001	0.042
CRP^b^	0.081	0.031–0.333	0.018	CRP^b^	0.083	0.039–0.335	0.013
NT-ProBNP	0.187	0.183–0.423	<0.001	NT-ProBNP	0.196	0.199–0.435	<0.001
b)							
D-dimer	0.063	<−0.001– < 0.001	<0.001	F1 + 2	0.024	<−0.001– < 0.001	0.446
Age	0.180	0.011–0.023	0.050	Age	0.190	0.012–0.024	<0.001
Sex	−0.083	−0.405– − 0.061	<0.001	Sex	−0.085	−0.412– − 0.067	0.007
BMI	−0.086	−0.039– − 0.006	0.008	BMI	−0.087	−0.040– − 0.006	0.008
HT	0.125	0.145–0.445	<0.001	HT	0.126	0.148–0.449	<0.001
Time frame^a^	0.307	0.031–0.046	<0.001	Time frame^a^	0.307	0.031–0.046	<0.001
CRP^b^	0.035	−0.059–0.217	0.261	CRP^b^	0.043	−0.041–0.233	0.169

Multivariable regression analysis adjusted for age, sex, BMI, HT, Time frame, CRP and NT-proBNP

For abbreviations, see text

^a^Time from symptoms to blood sampling ^b^Logtransformed ^c^ Confidence Intervals

### Prothrombotic markers and traditional risk factors

Levels of D-dimer and F1 + 2 were significantly higher in women (*p* < 0.001, both), while ETP was higher in men (*p* = 0.001) (Table 3). There were no significant sex differences in other CAT variables (data not shown).

No difference in any of the prothrombotic markers between smokers and non-smokers or patients with or without previous CVD was observed.

In patients with hypertension, D-dimer, F1 + 2 and velocity index levels were significantly higher compared to the group without hypertension (all *p* < 0.001), however the association weakened after adjustments for covariates (*p* = 0.018, *p* = 0.015 and *p* = 0.026, respectively).

Diabetic patients had significantly lower ETP levels compared to non diabetics (*p* = 0.004) without any differences in other CAT variables (data not shown) or D-dimer and F1 + 2. There were also limited correlations between the haemostatic markers and HbA1c and fasting glucose, except for velocity index which correlated weakly to fasting glucose (Table 2).

Significant inverse correlations were observed between BMI and D-dimer and F1 + 2 (both *p* < 0.001), whereas all CAT variables were positively correlated with BMI (all *p* < 0.05) (Table 2). There were significant trends for decreased levels of D-dimer and F1 + 2 and increased ETP across quartiles of BMI (adjusted *p* = 0.011, *p* = <0.001, *p* < 0.001, respectively).

## Discussion

In this large cohort of STEMI patients we found that levels of D-dimer and F1 + 2 were significantly associated with the extent of myocardial injury as measured by peak TnT. Significant associations between these coagulation markers and myocardial function, assessed by LVEF and NT-ProBNP, were further demonstrated.

We observed an inverse pattern for the *in vivo* thrombin generation and *ex vivo* potential to generate thrombin, which confirm previous findings in patients with stable CAD [16]. It might be speculated that this is due to an increased *in vivo* production of thrombin in the acute phase, resulting in reduced potential to generate thrombin *ex vivo*, as an exhaustion phenomenon.

Patients with STEMI admitted to primary PCI, receive heparin before or during the procedure. Heparin could potentially influence the results. However, as heparin was given only during the procedure, any effect on the measured variables was most likely not present when the blood samples were drawn 18 h (median time) after the procedure.

There was a clear association between the variables and myocardial necrosis measured by peak TnT. This association was also present after adjustments for potential covariates including CRP. Thus the prothrombotic state, to some degree also reflected in CAT parameters, was probably not a result of inflammation in the acute phase. We have previously reported similar results in another AMI population [17]. In that particular study the prothrombotic markers were measured 3–4 days after the acute event, probably reflecting a more stable situation. Nevertheless, the results clearly indicate that patients with larger infarctions are in an increased hypercoagulable state. It might therefore be discussed if patients with large infarctions are sufficiently protected by use of double antiplatelet therapy [18]. Use of warfarin has been shown to reduce both D-dimer and F1 + 2 after AMI [19], and randomized, clinical studies have shown beneficial effects on clinical outcome by use of warfarin as anticoagulation after acute MI [20, 21].

The findings of a significant association between procoagulant activity, shown especially by D-dimer and F1 + 2, but also by CAT variables, and impaired myocardial function in the acute phase of a STEMI, has to our knowledge, not been reported before. Elevated prothrombotic markers in the early phase of AMI are known to identify patients with incrased risk of subsequent cardiac death, but such associations have so far been reported to appear independent of LVEF [22]. Elevated levels of D-dimer and F1 + 2 were shown along with impaired myocardial function in another population not suffering from CAD [23]. An association between elevated D-dimer and heart failure has also been demonstrated [24]. Although there is no convincing evidence that oral anticoagulant therapy reduces mortality and vascular events in patients with heart failure and sinus rhythm [25, 26], prolonged anticoagulant treatment of such patients may be discussed after an AMI.

Diabetes is generally associated with elevated levels of prothrombotic markers [27], also in diabetic patients without coronary heart disease [28]. In our population, diabetes and glucometabolic disturbances were limited associated with a prothrombotic state, other than lower levels of ETP and a significant correlation between fasting glucose and velocity index. The latter may indicate glucose per se to play a role for the propagation phase of thrombin generation. Our results differ from some other studies showing enhanced thrombin generation in diabetics [28–30]. However, in the study by Tripodi et al. ETP levels were higher in diabetics versus controls only in the presence of added thrombomodulin [29]. Difference in the populations investigated may also be of importance. The limited findings in our study may be explained by the elevated levels of prothrombotic markers in the acute situation of an AMI, thus masking any difference. In addition, the levels of fasting glucose and HbA1C indicate adequate treatment of diabetes in the present population. Similar results have also been shown in another study on stable patients with CAD [16].

The inverse correlations between BMI and D-dimer and F1 + 2 indicating a less hypercoagulable state in overweight individuals, are in accordance with previous findings in a population of stable CAD patients [16] and is not easily explained. In contrast, all CAT variables were positively associated with BMI, indicative of an increased potential to thrombin generation. Other studies have shown positive correlation between BMI and D-dimer, however, only in patients not diagnosed with CVD [31]. In one study on healthy, obese individuals D-dimer values were found not to be correlated to BMI [32].

Increased levels of prothrombotic markers in patients with hypertension is well known [33–35]. This was also present in our population of STEMI patients when evaluated in the acute phase, showing elevated levels of D-dimer, F1 + 2 and velocity index in the group of hypertensive patients, although highly dependent of related factors.

### Limitations

Single bloodsampling prevented us from studying the time-course of the measured markers. We are not sure to have measured peak values of the variables or transient changes due to the variability in the time frame from onset of symptoms to blood sampling. However, the results did not change when taken this into account in the multivariate models. The blood samples were centrifuged at 4° C, thus any contact activation cannot be ruled out. We have also not included an extra centrifugation step before the analysis. The measure of LVEF by echo cardiography was performed at different time points from hospital discharge until 3 months after the index infarct, and our cohort of STEMI patients was a low risk population with few complications and just slightly reduced LVEF, and this fact may have influenced the results. As we do not have follow-up information of this cohort, any impact of the results on future clinical endpoints cannot be explored.

## Conclusion

In our cohort of STEMI patients we could demonstrate a significant association between levels of D-dimer and F1 + 2 and the extent of myocardial necrosis as assessed by TnT. The high levels of these markers in patients with low LVEF and high NT-ProBNP may indicate a hypercoagulable state in patients with impaired myocardial function. The inverse relation between BMI and procoagulant activity is not easily explainable, and has to be further explored.
